# G, N, and P Gene-based Analysis of Chandipura Viruses, India

**DOI:** 10.3201/eid1101.040602

**Published:** 2005-01

**Authors:** Arankalle Vidya Avinash, Shrotri Sandhya Prabhakar, Walimbe Atul Madhukar, Hanumaih, Shailesh Dattatraya Pawar, Mishra Akhilesh Chandra

**Affiliations:** * National Institute of Virology, Pune, Maharashtra, India

**Keywords:** Chandipura virus, Encephalitis, G (glycoprotein) gene, N (nucleocapsid) gene, P (phosphoprotein) gene, Phylogenetic analysis, dispatch

## Abstract

An encephalitis outbreak in 2003 in children from India was attributed to Chandipura virus. Sequence analyses of G, N, and P genes showed 95.6%–97.6% nucleotide identity with the 1965 isolate (G gene, 7–11 amino acid changes); N and P genes were highly conserved.

Chandipura virus (CHPV, family *Rhabdoviridae)*, was implicated as the cause of a large outbreak of encephalitis in children, involving 329 cases with 183 deaths, from Andhra Pradesh state, India in 2003 (*1*). On the basis of serologic investigations conducted during the epidemic, CHPV infection led to different clinical manifestations, including subclinical cases, mild fever, and encephalitis; some patients died within 48 hours, while others recovered (*1*). CHPV was described for the first time in India in 1965, when it was isolated from the serum of a patient with febrile illness (*2*) during an outbreak of dengue and Chikungunya viruses. The virus was isolated again in 1980 from an encephalopathy patient during an outbreak in children (*3*). However, the magnitude of the 2003 outbreak was unique. The present study was conducted to understand the relationship of the 2003 isolates with the 1965 strain and to assess association of mutations in G, N, and P genes with different clinical manifestations.

## The Study

During the outbreak investigations, 5 CHPV isolates were obtained in cell culture. Table 1 provides details about these isolates. The 1980 isolate was not available for further analysis. These isolates were subjected to reverse transcription–polymerase chain reaction (RT-PCR), according to the previously described method (*1*). The primers listed in Table 2 were designed on the basis of published sequences and used to amplify and sequence the G, P, and N genes (*4*,*5*). The PCR products were purified by using Wizard PCR preps DNA purification Kit (Promega, Madison,WI) and sequenced by using Big Dye Terminator cycle sequencing Ready Reaction Kit (Applied Biosystems, Foster City, CA) and an automatic sequencer (ABI PRISM 310 Genetic Analyzer, Applied Biosystems).

**Table 1:** Details of the Chandipura viral isolates examined

Patient no	Place (state)	Isolate/date of origin	Cell line	Inoculum	Clinical category	Accession no.
1	KarimNagar (AP)*	CIN0327M July 2003	MDCK	Throat swab	Encephalitis	G gene: AY382603 N/P gene: AY614725
1	Karimnagar (AP)	CIN0327R July 2003	RD	Throat swab	Encephalitis	G gene: AY614718 N/P gene: AY614726
2	Karimnagar (AP)	CIN0360R July 2003	RD	Brain	Encephalitis	G gene: AY614719 N/P gene: AY614731
2	Karimnagar (AP)	CIN0360V July 2003	Vero	Brain	Encephalitis	G gene: AY614720 N/P gene: AY614730
3	Karimnagar (AP)	CIN0331M July 2003	MDCK	Throat swab	Encephalitis	G gene: AY614721 N/P gene: AY614729
4	Karimnagar (AP)	CIN0309R July 2003	RD	Throat swab	Fever	G gene: AY614723 N/P gene: AY614728
5	Karimnagar (AP)	CIN0318R July 2003	RD	Throat swab	Fever	G gene: AY614722 N/P gene: AY614727
6	Chandipura (Maharashtra)	CIN6514V† June1965	BS-C-1	Serum	Fever	G gene: AY614717 N/P gene: AY614724

*AP, Andhra Pradesh †1965 isolate.

**Table 2:** Primers used for amplification and sequencing

Gene	Primers
G gene	
CHAND-G-F1	27-5′ ATGACTTCTTCAGTGACAATTAGT 3′-50
CHAND-G-F2	425-5′ GTCTTGTGGTTATGCTTCTGT 3′-445
CHAND-G-F3	853-5′ TGTGTCCGACCGGGATCAGAGGT 3′-875
CHAND-G-F4	1278-5′ GACAATGAACTACACGAGCT 3′-1297
CHAND-G-R1	1741-5′ TCATCCACCGGGTTGAGATCCAT 3′-1708
CHAND-G-R2	1342-5′ TGAGCATGAGGTAGCTGTGGAT 3′-1321
CHAND-G-R3	30-5′ TCCTCTGAATCTCTGAGGTC 3′-911
CHAND-G-R4	471-5′ TGATTACCAAGAACTCAGAGT 3′-451
N / P gene	
CHAND-N-F1	31-5′ TATAGTAGTACACGAACACT 3′-50
CHAND-N-F2	481-5′ TCTTTGGTCTTTATCGTG TGT 3′-501
CHAND-N F3	871-5′ TTGACCAAGCTGATTCCTACAT 3′-892
CHAND-N-F4	1279-5′ TAGGAGATATTCGAGTGAACT 3′-1299
CHAND-N-F5	1742-5′ TGAGTGCTCTCCAACTTCTGCAGT 3′-1765
CHAND-N-F6	2281-5′ CAGATTCTCTGTTGCTTACCACT 3′-2306
CHAND-N-R1	531-5′ TCTTCTTGTACTCGACCTGT 3′-512
CHAND-N-R2	942-5′ TTGAAGAGTAAGGAGACTTCGT 3′-921
CHAND-N-R3	1320-5′ TCCTGGCGTACTCTGCAACT 3′-1301
CHAND-N-R4	1830-5′ TGTGCTGATCTGCAACAGCCT 3′-1810
CHAND-N-R5	2331-5′ TTCTTCAGAGCTTGCATCTTGAT 3′-2309
CHAND-3′-F	11-5′ TATGTCTTATAAGAATGCTATT 3′-32

Multiple alignment of nucleotide/amino acid sequences was carried out by using software ClustalX v.1.83. Phylogenetic analyses based on the G, N, and P genes (1593, 1269, 882 nucleotides [nt], respectively) were carried out employing maximum likelihood method in Phylo_win software (*6*). The reliability of different phylogenetic groupings was evaluated by using the bootstrap test, with 1,000 bootstrap replications, available in Phylo_win. CHPV sequences representing 3 encephalitis cases, including 1 fatal case (patient 2, Table) and 2 febrile cases, were compared.

G gene analysis led to the correction of the sequence reported for the 1965 isolate (accession no. J04350). As compared to the 1965 isolate, the only sequence available in the GenBank database, the following differences were noted for all the 2003 epidemic isolates: 1) an addition of 17 nt after position 1457 base; 2) additions at positions 804, 902, and 1558; and 3) deletions at positions 854 and 869. To confirm these mutations, we sequenced the 1965 isolate available with the repository of the institute and noted that the 1965 sequence did not exhibit the deletions or additions mentioned above. The corrected 1965-CHP-G gene sequence was deposited in GenBank (accession no. AY614717) and used for comparisons. When compared with the corrected sequence, the 2003 epidemic isolates did not exhibit the mutations mentioned above. Although Walker and Kongsuwan resequenced part of the G gene of the 1965 isolate (262 nt) and made necessary corrections (*7*), these were not deposited in GenBank.

**Figure 1:**
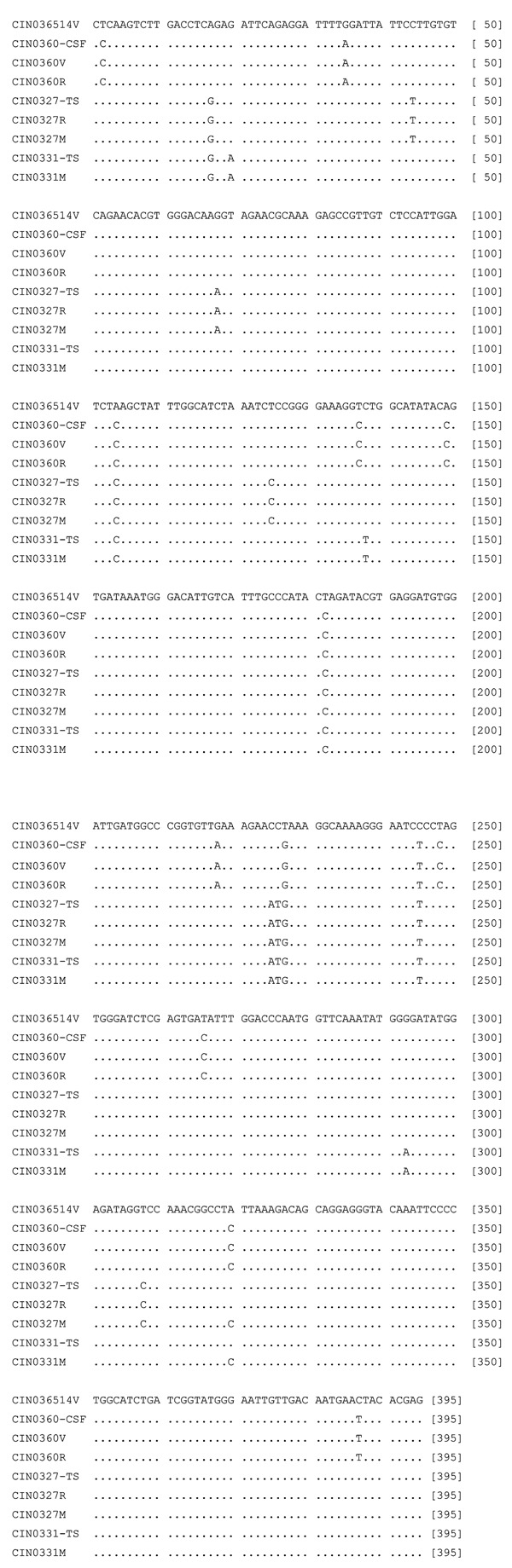
Partial G gene nucleotide sequence alignment of 3 Chandipura viruses (CHPV) isolated during the outbreak along with the corresponding sequence derived from the clinical samples. For details on isolates, see Table 1. GenBank accession numbers for the sequences derived from clinical specimens and published earlier (*1*) are AY554407, AY554409, and AY554411.

The epidemic isolates exhibited 97% ± 0.3% nucleotide identity (PNI) with each other and 95.6%–96.1% PNI with the 1965 isolate. For CIN0360 and CIN0327 isolates, grown in 2 different cell lines, the PNIs were 100% and 99.9%, respectively. Comparison of partial G gene sequences from clinical samples (N = 3) with the corresponding cell-line isolates documented that, although sequences derived from different clinical samples exhibited unique mutations, except for l substitution in CIN0331M isolate (A1167C), no changes were noted (see Figure 1).

**Figure 2:**
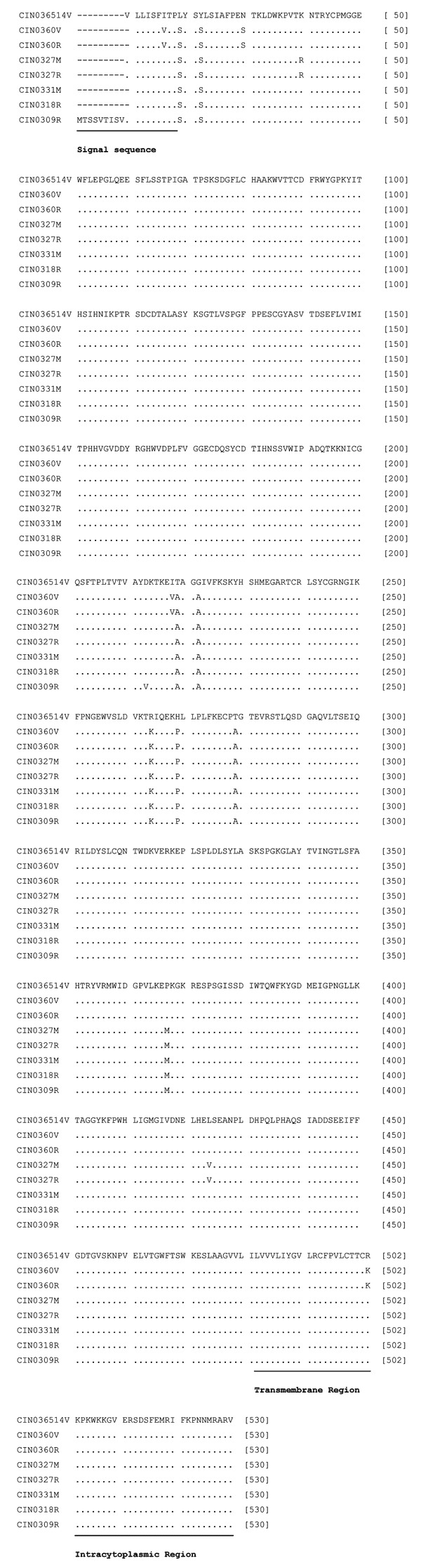
Alignment of the deduced amino acid sequences of the G protein of different isolates of Chandipura virus. For details on isolates, see Table 1. Solid bars represent signal sequence (1–18 amino acids [aa]), transmembrane region sequence (482–502 aa), and the intracytoplasmic region sequence (503–530 aa).

Alignment of deduced amino acid sequences of the G protein (530 amino acids [aa]) from different isolates is depicted in Figure 2). A total of 7 aa substitutions were noted for the epidemic isolates: Leu19Ser, Tyr22Ser, Thr219Ala, Gly222Ala, Arg264Lys, His269Pro, and Thr279Ala. In addition, the brain-derived isolate exhibited 4 more substitutions: Ile16Val, Asn30Ser, Ile218Val, and Arg502Lys. This isolate did not replace Pro → Met at position 367 seen in other epidemic isolates. Two amino acid substitutions (Lys40Arg and Leu424Val) were seen in the isolates from encephalitis cases (CIN0327M and CIN0327R). One isolate from a febrile patient, CIN0309R, showed an additional substitution, Asp213Val.

**Figure 3:**
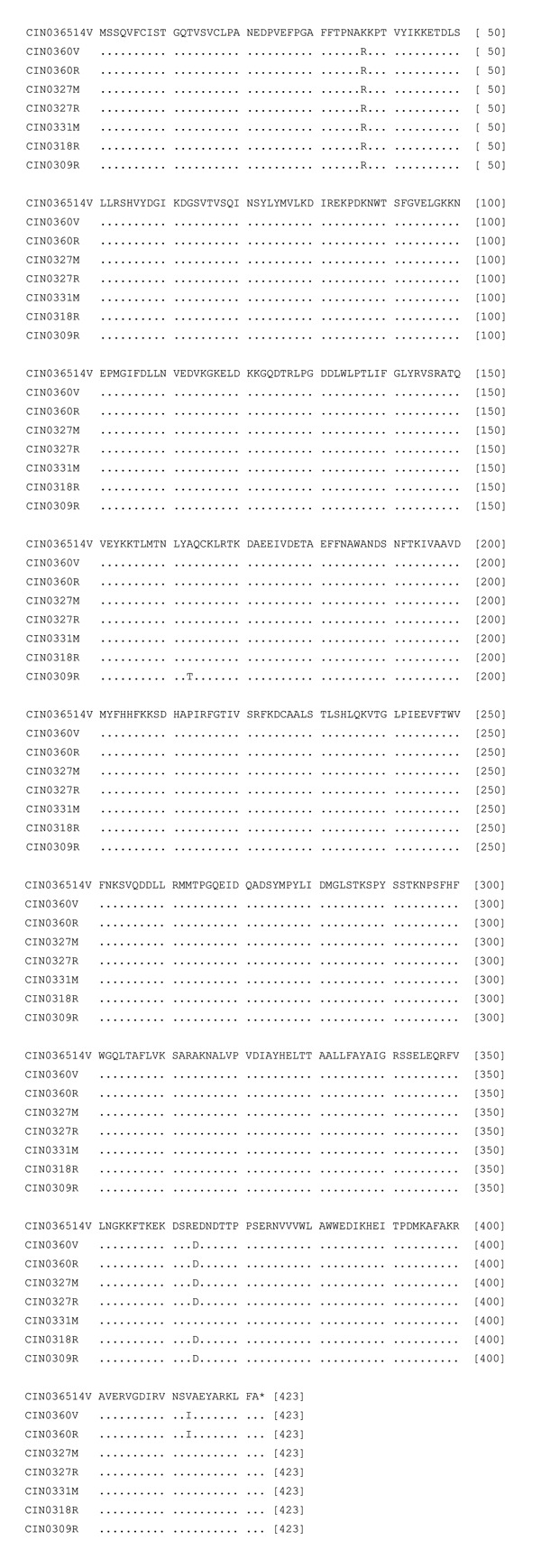
Alignment of the deduced amino acid sequences of the N protein of different isolates of Chandipura virus. For details on isolates, see Table 1.

N gene analysis showed that the 1965 isolate was 96.5%–97.6% identical at the nucleotide level with the epidemic isolates, whereas the epidemic isolates were 97.7% ± 0.3% identical with each other. The isolates grown in different cell lines exhibited 99.3%–99.5% PNI. A single amino acid substitution, Lys37Arg, was noted for all epidemic isolates (Figure 3). In all isolates except CIN0331M, Asp substituted Glu at 364. Additional substitutions, Val413Ile (brain-derived isolate) and Ala163Thr (CIN0309R, from a febrile case), were present.

**Figure 4:**
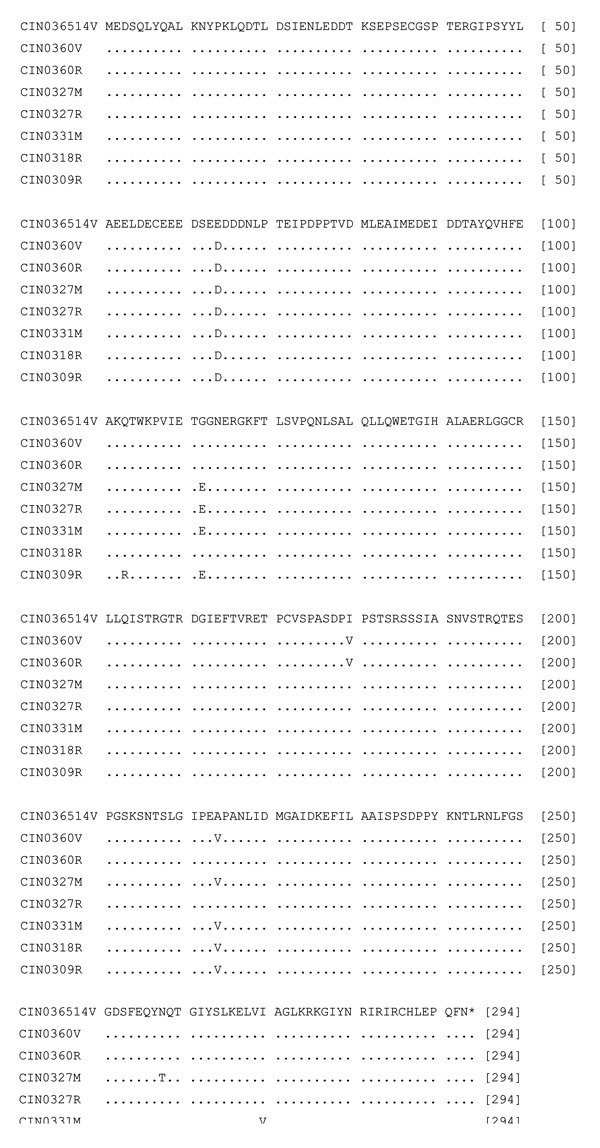
Alignment of the deduced amino acid sequences of the P protein of different isolates of Chandipura virus. For details on isolates, see Table 1.

For the P gene, among epidemic isolates, the PNI was 97.4% ± 0.4%, whereas 95.8%–96.8% identity was observed with the 1965 isolate. The isolates grown in different cell lines were 99%–99.7% identical at the nucleotide level. Glu64Asp substitution was present in all the epidemic isolates (Figure 4). A unique single amino acid substitution was noted for 3 isolates: Gln103Arg in CIN0309R (febrile case), Ile180Val in brain-derived isolates, and Asn257Thr in CIN0327M (encephalitis case). In addition, Gly112Glu substitution was recorded in 4 isolates (CIN0327R, CIN0327M, CIN0309R, and CIN0331M); Ala214Val was present in all except CIN0327R, CIN0360R, and Ile270Val in 3 isolates (CIN0318R, CIN0309R, and CIN0331M).

**Figure 5:**
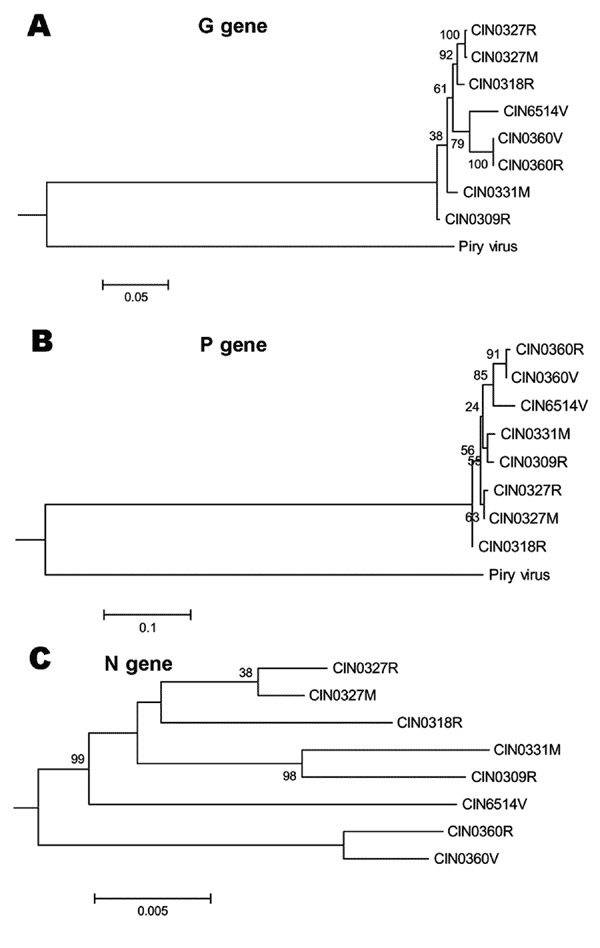
Phylogenetic analyses of complete G gene (A), P gene (B), and N gene (C) of Chandipura virus isolates. For details on isolates, see Table 2. Percent bootstrap support is indicated by the values at each node. For G and P gene-based analyses, Piry virus (GenBank accession no. D26175) was used as an outgroup. For N gene, an unrooted tree was constructed because the sequence for Piry virus was not available.

The Figure 5 presents the phylogenetic status of different epidemic isolates. Overall, different CHPV isolates were not very divergent from each other. For G and P gene–based analyses, the brain-derived isolate was closer to the 1965 isolate. No segregation of fever and encephalitis case-derived isolates was noted. Although the topology for the unrooted N gene–based tree was similar, the 1965 isolate remained on a separate branch.

## Conclusions

This study showed that the 2003 epidemic isolates were closely related to the 1965 isolate. PNIs were 95.6%–96.1% for the G gene, which is responsible for virus entry into cells and induction of neutralizing antibodies; 96.5%–97.6% for the N gene, mainly associated with cytotoxic T-lymphocyte responses; and 95.8%–96.8% for the P gene, associated with RNA polymerase. Thus, the epidemic was not associated with extensive mutations in these genes. Adaptation to cell cultures did not result in changes in the partial G gene sequences, except for l nt change (A to C at position 1167) for 1 isolate.

The comparison of the deduced amino acid sequences of G protein of 1965 and 2003 isolates documented 7 differences for the epidemic isolates. None of these were in the transmembrane region sequence (482–502 aa) or in the intracytoplasmic region sequence (503–530, the carboxyl end of the protein). No change in the signal sequence was noted for CIN0309R, the only isolate sequenced completely in this region. Additional amino acid substitutions were recorded for the brain-derived isolate. These included Ile16Val, the signal sequence, and Arg502Lys, the transmembrane region sequence. Both N and P proteins were highly conserved, with only 1 aa substitution at positions 37 and 64, respectively. Importance of the amino acid substitutions in these proteins in the pathogenesis of CHPV infection remains to be determined. As modeled by Walker and Kongsuwan (*7*), major antigenic sites for Vesicular stomatitis virus (New Jersey) neutralization escape mutations correspond to the CHPV G domain exhibiting multiple amino acid changes in epitope VII (Thr219Ala and Gly222Ala) and epitope VI (Arg264Lys and His269Pro).

Phylogenetic analyses based on G and P genes (Figure 5) showed that the brain-derived isolate clustered with the 1965 isolate. No segregation of the isolates from encephalitis and febrile cases was noted, regardless of the type of the viral gene examined, a finding that suggests the importance of host factors in influencing the outcome of the infection.

In conclusion, the present study shows that Chandipura viruses isolated from human cases in India in 1965 and 2003 were not very divergent. Although several amino acid differences were recorded in G protein, the importance of these changes in the pathogenesis of CHPV infection needs to be determined. Generation of infectious cDNA clones for 1965 and 2003 isolates and assessment of individual genes in the pathogenesis of CHPV infection may help in understanding the relationship of structure to outcome for CHPV infections.
